# The Recent National Conventions

**Published:** 1894-09

**Authors:** 


### Editorial.

### THE RECENT NATIONAL CONVENTIONS.

The annual conventions having now completed their labors, a brief *résumé* of the work accomplished may be of value in advance of detailed reports.

The Southern Dental Association met August 2, at Old Point Comfort, followed by the National Association of Dental Faculties on August 4, and subsequently by the National Association of Dental Examiners, American Dental Association, and the School of Dental Technics.

It was not the privilege of the writer to attend but one session of the Southern, but that was fully occupied with interesting subjects for thought and discussion and exhibited an earnest effort to make this annual convocation worthy the best intelligence of the South.

The two important bodies, the National Association of Dental Faculties and the National Association of Dental Examiners, secured more of the interest of educators in attendance, as it was anticipated some important changes would be introduced, especially in the first-named body.

The "Faculties" is always an active centre of thought, for here must be arranged plans for the future development of dental education in this country, and upon its action depends the character of the entire dental educational system of the United States. The responsibility is great, but it has been met with an intelligent appreciation of the needs of the profession.

The impression that seems to prevail in some quarters that this organization has assumed the character of a "trades union society," selfishly working for the interest of the colleges united with it, is a mistaken opinion. It was originally formed to advance dental education, and has at no time swerved from this idea. The great advances made in this direction must be accredited to this body, for every move made in the interest of progress has had its inception in this organization, and was not the result of outside pressure.

The principal object of interest in this meeting, beyond routine business, was the effort made to bring all the schools in harmony as to time. This was accomplished by the adoption of a resolution requiring all colleges belonging to this organization to extend the regular winter session to not less than seven months, to take effect in 1896–97.

Another important rule adopted was that no school can be recognized unless a member in good standing with this body. Heretofore it has been the custom of some schools to give credit for time without regard to membership. This will force all newly organized colleges to take steps to secure admission to the Association. The latter, however, now demands the endorsement of the State Examining Board before it can be placed on the list of applicants.

There was a strong and very proper effort made to advance the preliminary examination so that, in addition to an English education up to the standard required for entrance to the high schools, there should be demanded Latin and physics. The colleges were very nearly equally divided for and against this proposition. The opposition argued that the time had not yet arrived to take this advanced step, and it could not safely be made until the grammar schools of the country advanced their standard. Until this were done it would necessarily keep out of the profession many worthy young men. It was also contended that the organization should not take a step forward it could not maintain, and this must be the result in the present condition of education in this country. The matter was, on reconsideration, having passed at a previous session, laid on the table. It is recognized that this advance must eventually be made, but it must come in the natural order of development.

A resolution demanding four years for the course of study was introduced and laid over for action next year. It contemplates commencing this advance in 1898–99. It is hoped that the century will close with all these reforms perfected.

The National Association of Dental Examiners is a body somewhat apart and naturally, from the character of its work, more exclusive than the last-named body, but is equally as important in its relations to dental education. For details of its work our readers must scan the published reports.

The "School of Dental Technics," a newly organized body, also held several sessions, to complete the organization commenced last year. This is a child of Western energy. As far as understood by the writer, it aims to improve the methods of teaching operative and mechanical dentistry. It is proposed that each college shall send a delegate to the annual meeting to be held at the same time and place as that decided upon by the National Association of Dental Faculties. The effort is a laudable one, and the indications are that its work will have good results, more especially in recently organized schools where the chairs are filled by inexperienced men.

The American Dental Association convened August 7. About one hundred and fifty members and delegates were present. The story of this meeting had perhaps better be left for the reports. From a scientific view it was the most unsatisfactory meeting the writer has attended in many years, while, on the other hand, it was socially a decided success. There were several excellent papers and reports, but the sections generally seemed to have either hastily prepared their work or neglected it altogether, and as a result the proceedings were hardly above the standard of an interior local society.

The Association was unfortunate in selecting Old Point Comfort as a place of meeting. It proved the reverse of comfortable; in fact, the room in which the meetings were held was so intensely hot that to deliberate in such an atmosphere became a torture, and the result was a poor attendance and general loss of interest. While the surroundings of Fortress Monroe are full of interest and the panorama of a most varied character, it is not the place for a deliberative body to meet in mid-summer, and should be avoided in the future.

Two important matters were considered at this meeting, and both were left in the hands of committees. The first was a union of the Southern and American Associations. This has been long desired, but up to the present time seemed impossible of attainment. Now it appears not only possible, but certain of being accomplished before the expiration of another year. In view of the certainty of this, the American Association postponed action on the report of the Committee on Revision of the Constitution, so that the Southern members could have an opportunity of expressing their opinions and voting for the laws to govern the united body.

It is to be hoped that when this document is finally placed as the law of the reorganized body, it will have been so framed that members must work to maintain the scientific character of the organization. If this be not done, the days of the American Dental Association are numbered. It is trusted, however, that in the reorganization wise counsels may prevail and that the hallowed memories clustering round this body will not be permitted to die, but that in the infusion of new life it may advance steadily towards scientific methods in its future meetings.

Asbury Park was selected as the place of the meeting in 1895. That the objections to Old Point Comfort will not apply to this place the members may be assured. Everything is there to satisfy the most exacting. Our objection is that it is too far East and that the Association should have selected some place more centrally located.

